# In vitro evaluation of the effects of different particle types in toothpastes on the efficacy against enamel erosion and wear

**DOI:** 10.1038/s41598-022-13922-7

**Published:** 2022-06-10

**Authors:** Melanie Fischer, Nadine Schlueter, Stefan Rupf, Carolina Ganss

**Affiliations:** 1 Department of Conservative and Preventive Dentistry, Dental Clinic of the Justus-Liebig-University Giessen, Giessen, Germany; 2 Division for Cariology, Department of Operative Dentistry and Periodontology, Center for Dental Medicine, Medical Center, Faculty of Medicine, University of Freiburg, Freiburg, Germany; 3 Department of Conservative Dentistry, Periodontology and Preventive Dentistry, Hannover Medical School, Hannover, Germany; 4 Clinic of Operative Dentistry, Periodontology and Preventive Dentistry, Chair of Synoptic Dentistry, Saarland University, Homburg, Germany

**Keywords:** Health care, Medical research

## Abstract

The effects of the particle fraction in toothpastes in the context of erosion and erosive tooth wear has not been fully elucidated. Thus, aim of this study was to investigate experimental toothpastes, each with one specific particle type. Toothpastes with seven different types of silica or alumina were prepared as slurry either with or without active ingredients (NaF or F/Sn). Human enamel samples were exposed to a cyclic erosion/abrasion model, and were either treated with the respective slurries only or additionally brushed in a brushing machine. Tissue loss was profilometrically monitored. After treatment with slurries without active ingredients or with NaF, tissue loss increased significantly within groups over time (*p* < 0.001 each). At the end of the trial, there were minor differences between groups (not exceeding 10–20%; *p* > 0.05 for most comparisons). After treatment with the F/Sn slurries, tissue loss stagnated completely over time, with the exception of one silica type and alumina, but both still reduced tissue loss by 40–50% (compared to control *p* < 0.001 each). Relative to the type of the active ingredient, the particle type seems to be a secondary factor for the efficacy of toothpastes on erosion and erosive tooth wear in enamel.

Toothpastes are ideal carriers for active substances against various diseases of the oral cavity. At the same time, they are one of the most widely used products of daily oral hygiene. Thus, toothpastes are meanwhile marketed for a wide range of specific indications, including the prevention of erosive tooth wear.

The efficacy of toothpastes for erosion prevention can vary considerably for the same active ingredient as well as for combinations of active ingredients^1^. In vitro experiments that have investigated the effects of active substances in toothpastes and used toothpaste slurries without additional brushing abrasion for this purpose show effect sizes ranging from no effect to a reduction in substance loss of up to 50%^2,3^. Toothpastes containing stannous ions are more difficult to compare because they not only contain different stannous compounds but also different concentrations of stannous ions. They generally have revealed somewhat better results than toothpastes without stannous ions^1^, but even these products have different degrees of efficacy.

For daily oral hygiene, toothpastes are used in conjunction with toothbrushing, so that a physical component always plays a role as well. Thus, there is usually a slightly higher tissue loss compared to when used alone as a slurry, but this effect can also vary greatly between different market products. While brushing has no or only a minor adverse effect with some products, tissue loss can be substantially increased with others. This means that a promising active ingredient effect can apparently be counteracted by the particle fraction^1^. It seems therefore reasonable to assume that the particle fraction in the toothpaste formulations plays an important role. These particles serve on one hand as thickeners so that the toothpaste achieves a suitable consistency, but they are also intended to improve tooth cleaning through their abrasiveness. To fulfil these different functions, there are particles of different sizes and types, such as silica, hydrated silica, alumina or calcium carbonate available. Such particles also have different hardness and shape^4^.

The significance of the particle fraction in the context of erosive tooth wear has been little studied so far. First of all, it is assumed that products with high abrasiveness in terms of REA (relative enamel abrasion) and RDA (relative dentin abrasion) cause more enamel loss than products with low REA/RDA values. This has at least partly been shown for experimental toothpastes without active ingredients^5,6^; if active ingredients are added to the formulations, this association is, however, attenuated^6,7^ or very weak^3,8^. Further, particles may not only interact with the tooth surface, but also with the ingredients of toothpaste formulations. An example is the finding that tin can adsorb in varying amounts on particle surfaces^9^, thus potentially reducing the efficacy of respective toothpastes.

Only a few studies have explored this particle fraction in more detail in the context of dental erosion^3,10^, and the results of these studies are inconclusive so far. Since the market products used in the abovementioned experiments contained mixtures of different particle types, it is difficult to draw any conclusions regarding the effects of individual particle types.

Therefore, the aim of the present study was to investigate whether specific particle types can influence the efficacy of toothpastes against erosion/abrasion in different ways. For this purpose, experimental toothpastes with only one particle type each and otherwise identical formulation were investigated both without and with different active ingredients. The impact on erosive and erosive-abrasive tissue loss was monitored over four time points (T1 to T4).

The null hypothesis was that different particle types in experimental toothpastes had no effect on tissue loss values when applied as slurries only and no effect on tissue loss values when applied as slurries with additional brushing, regardless of active ingredients.

## Results

### Application of slurries without brushing abrasion

#### Within group comparisons

In the control groups, there was a continuous increase in tissue loss from the first to the last measurement time point (T1 to T4; p at one time point to the respective previous time point < 0.001 each). Regardless of the particle type, this was also the case after application of the NaF slurries. After application of the F/Sn slurries, however, this pattern was only seen in the less effective slurries with Apyral and Sylodent (T1 to T4; p at one time point to the respective previous time point each < 0.001). In the other groups, no significant increase was found after an initial loss (T2 to T1, T3 to T2 and T4 to T3: *p* > 0.002 each). Data are given in Table 1.

**Table 1:** Tissue loss values in µm (mean ± SD) after application of slurry only as well as after additional brushing for all time points. Asterisks indicate no significant difference to the time point before (level of significance .002 after Bonferroni adjustment).

	Slurry only				Slurry + brushing			
	T1	T2	T3	T4	T1	T2	T3	T4
**No active ingredients (series I)**								
Control					6.0 ± 1.4	12.9 ± 2.0	19.0 ± 2.1	25.4 ± 2.4
Apyral					5.8 ± 0.6	12.7 ± 1.0	18.6 ± 2.2	25.6 ± 1.7
Sylodent					5.3 ± 0.6	11.7 ± 0.5	17.7 ± 1.2	23.8 ± 1.2
Syloblanc					5.5 ± 1.0	12.7 ± 1.2	18.9 ± 2.0	25.5 ± 2.7
Perkasil					5.4 ± 0.8	12.2 ± 1.3	18.9 ± 1.4	25.9 ± 1.8
Sorbosil 77					5.4 ± 1.0	12.1 ± 0.8	17.5 ± 1.4	23.5 ± 1.7
Sorbosil 36					4.7 ± 0.7	12.2 ± 0.9	18.5 ± 1.0	25.0 ± 1.3
Sorbosil 39					5.4 ± 0.8	13.1 ± 1.1	18.9 ± 1.4	25.0 ± 2.0
**NaF (series II)**								
Control	4.0 ± 1.0	9.6 ± 1.4	15.7 ± 1.7	18.8 ± 1.6	3.2 ± 0.7	9.8 ± 1.9	16.1 ± 1.4	22.6 ± 1.6
Apyral	3.4 ± 1.5	7.4 ± 2.5	12.5 ± 2.6	17.7 ± 2.7	2.1 ± 0.9	7.9 ± 1.2	13.5 ± 1.2	18.8 ± 1.2
Sylodent	2.4 ± 1.3	5.9 ± 2.4	10.1 ± 2.5	14.7 ± 2.5	3.5 ± 0.7	8.4 ± 0.8	13.0 ± 1.2	17.6 ± 1.5
Syloblanc	3.2 ± 1.3	7.3 ± 2.3	12.3 ± 2.7	16.7 ± 3.5	4.3 ± 0.6	9.4 ± 1.0	14.2 ± 1.7	18.6 ± 2.3
Perkasil	3.8 ± 1.5	8.0 ± 1.9	13.0 ± 1.5	17.6 ± 1.7	3.5 ± 0.7	8.6 ± 0.9	13.3 ± 1.0	18.1 ± 1.1
Sorbosil 77	2.9 ± 1.4	7.3 ± 1.8	12.0 ± 1.6	17.0 ± 1.9	3.6 ± 0.7	7.9 ± 0.9	12.3 ± 0.9	15.9 ± 1.4
Sorbosil 36	3.6 ± 2.1	8.0 ± 2.5	13.6 ± 2.1	18.2 ± 2.2	3.8 ± 0.6	8.7 ± 0.8	13.3 ± 0.8	18.1 ± 0.9
Sorbosil 39	4.3 ± 1.7	8.8 ± 2.2	13.2 ± 2.0	18.3 ± 2.0	3.1 ± 0.7	7.1 ± 1.6	12.8 ± 1.1	17.8 ± 1.3
**F/Sn (series III)**								
Control	5.1 ± 1.4	11.5 ± 1.4	17.6 ± 2.4	22.6 ± 2.8	6.3 ± 0.8	13.3 ± 1.1	20.2 ± 1.7	27.0 ± 2.0
Apyral	3.2 ± 0.9	5.1 ± 1.0	9.0 ± 2.4	10.5 ± 3.0	3.7 ± 0.6	7.6 ± 1.2	11.9 ± 1.7	15.7 ± 2.1
Sylodent	2.2 ± 0.7	4.3 ± 1.3	6.6 ± 1.6	9.2 ± 1.7	3.2 ± 0.7	7.0 ± 1.1	10.3 ± 2.0	13.3 ± 2.4
Syloblanc	2.2 ± 0.8	2.5 ± 1.3 *	2.4 ± 1.3 *	2.3 ± 1.3 *	2.3 ± 0.7	3.1 ± 0.9	3.6 ± 1.2	3.8 ± 1.1 *
Perkasil	2.1 ± 0.5	2.8 ± 0.7 *	3.5 ± 1.3 *	3.7 ± 1.6 *	2.3 ± 0.5	2.7 ± 0.9	2.9 ± 1.1	3.0 ± 1.0
Sorbosil 77	1.7 ± 0.8	2.0 ± 1.1 *	1.9 ± 1.2 *	1.8 ± 1.2 *	2.8 ± 0.7	3.0 ± 0.8	3.2 ± 0.9	3.2 ± 1.0
Sorbosil 36	1.7 ± 0.8	1.6 ± 0.9 *	1.9 ± 1.2 *	1.6 ± 1.2 *	2.1 ± 0.5	2.9 ± 0.4	3.3 ± 0.6	3.2 ± 0.6 *
Sorbosil 39	2.1 ± 0.7	2.3 ± 1.2 *	2.5 ± 1.6 *	2.9 ± 1.6 *	2.1 ± 0.3	2.6 ± 0.6	3.1 ± 0.8	3.1 ± 0.7 *

#### Between group comparisons

The overall effect of NaF was limited. Compared to the control group, tissue loss was only significantly reduced when the slurry with Sylodent was applied. This slurry showed significantly better results than those with Perkasil, Sorbosil 36, and Sorbosil 39.

In contrast, the effects of the F/Sn slurries were very substantial. Compared to the control group, tissue loss was significantly reduced in all test groups. However, slurries with Apyral and Sylodent showed less beneficial effects than the slurries with the other particle types. There were only minor differences between the five slurries with the best efficacy, the slurries with Sorbosil 77 and Sorbosil 36 were both significantly more effective than Perkasil, otherwise no significant differences were found.

**Figure 1:**
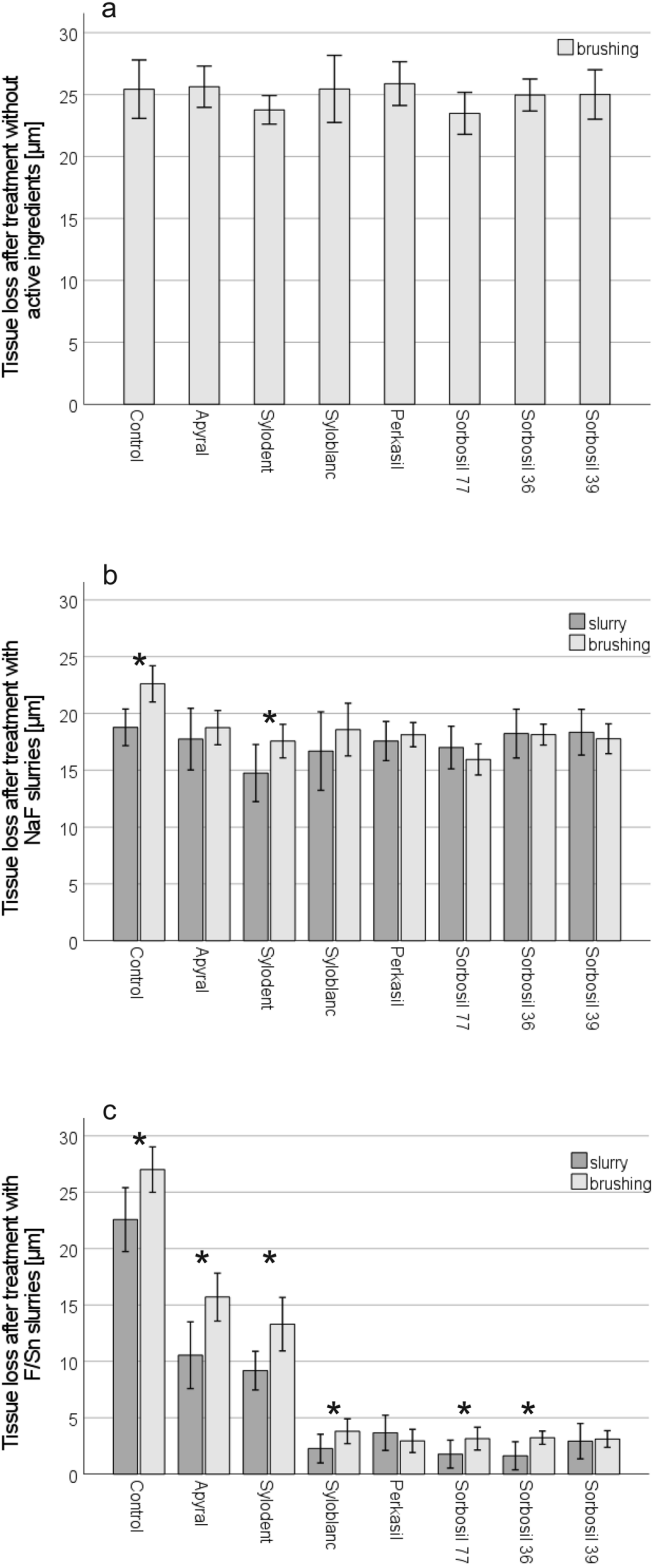
Tissue loss (mean ± SD) at the end of the experiment (T4) after treatment with slurries containing one of the various particle types each or control (distilled water). Slurries were prepared either without active ingredients (**a**), with NaF (**b**) or with F/Sn (**c**). Asterisks indicate a significant difference in tissue loss after slurry alone compared to additional brushing (level of significance .006 after Bonferroni adjustment). Significances between the particle types after treatment with the slurries only (dark grey columns) or after additional brushing (light grey columns) are shown in Table 2.

Tissue loss values at the end of the experiment (T4) after slurry application are given in Table 1 and Fig. 1; *p* values for all comparisons between particle types are given in Table 2.

**Table 2:** Compilation of *p* values (ANOVA) for comparisons of the effects of the different particle types at T4; significant differences are marked in bold.

	Apyral	Sylodent	Syloblanc	Perkasil	Sorbosil 77	Sorbosil 36	Sorbosil 39
**Brushing without active ingredients**							
Control	1.000	0.495	1.000	1.000	0.360	1.000	1.000
Apyral	–	**0.036**	1.000	1.000	**0.039**	1.000	1.000
Sylodent		–	0.700	**0.016**	1.000	0.295	0.783
Syloblanc			–	1.000	0.563	1.000	1.000
Perkasil				–	**0.017**	0.981	1.000
Sorbosil 77					–	0.296	0.631
Sorbosil 36						–	1.000
**NaF slurry only**							
Control	0.998	**0.000**	0.682	0.759	0.190	1.000	1.000
Apyral	–	0.081	1.000	1.000	1.000	1.000	1.000
Sylodent		–	0.906	**0.028**	0.200	**0.008**	**0.003**
Syloblanc			–	1.000	1.000	0.988	0.962
Perkasil				–	1.000	1.000	1.000
Sorbosil 77					–	0.951	0.819
Sorbosil 36						–	1.000
**NaF slurry + brushing**							
Control	**0.000**	**0.000**	**0.000**	**0.000**	**0.000**	**0.000**	**0.000**
Apyral	–	0.603	1.000	0.997	**0.000**	0.996	0.822
Sylodent		–	0.991	0.999	0.086	0.998	1.000
Syloblanc			–	1.000	**0.018**	1.000	1.000
Perkasil				–	**0.001**	1.000	1.000
Sorbosil 77					–	**0.000**	**0.016**
Sorbosil 36						–	1.000
**F/Sn slurry only**							
Control	**0.000**	**0.000**	**0.000**	**0.000**	**0.000**	**0.000**	**0.000**
Apyral	–	0.982	**0.000**	**0.000**	**0.000**	**0.000**	**0.000**
Sylodent		–	**0.000**	**0.000**	**0.000**	**0.000**	**0.000**
Syloblanc			–	0.245	1.000	0.995	0.999
Perkasil				–	**0.020**	**0.011**	0.997
Sorbosil 77					–	1.000	0.565
Sorbosil 36						–	0.367
**F/Sn slurry + brushing**							
Control	**0.000**	**0.000**	**0.000**	**0.000**	**0.000**	**0.000**	**0.000**
Apyral	–	0.135	**0.000**	**0.000**	**0.000**	**0.000**	**0.000**
Sylodent		–	**0.000**	**0.000**	**0.000**	**0.000**	**0.000**
Syloblanc			–	0.567	0.928	0.901	0.742
Perkasil				–	1.000	1.000	1.000
Sorbosil 77					–	1.000	1.000
Sorbosil 36						–	1.000

### Application of slurries with additional brushing abrasion

#### Within group comparisons

In the experimental series without active ingredients, brushing in the control group as well as brushing with the different particle containing slurries resulted in a continuous increase in tissue loss that was significant compared to the respective previous time point (each *p* < 0.001).

Regarding the groups treated with NaF slurries, the results corresponded to those of the application of the slurries without brushing abrasion. This was also the case for treatments with F/Sn slurries, except for a slight further increase in tissue loss until T3 when brushing with slurries containing Syloblanc, Sorbosil 36, and Sorbosil 39 (each *p* < 0.003 to the respective previous time point). Data are given in Table 1.

#### Between group comparisons

In the experiment without active ingredients, no significant differences were found between the slurries with the different particle types and the control group. Among the effects of the different slurries with particles, some significant differences were present, but these were only of minor order.

NaF significantly reduced the tissue loss in all groups compared to the control group, but the effect size was only minor. Brushing with Sorbosil 77 resulted in the numerically lowest tissue loss and was significantly lower than that of all other slurries except for Sylodent. In general, tissue loss did not increase with brushing compared to the application of the slurries alone, except for the slurry with Sylodent (*p* < 0.0001 compared to slurry application without brushing).

In contrast to NaF, F/Sn led to a very substantial reduction in tissue loss compared to the control group. Similar to the results with the application of the slurries without brushing, the least favourable results were found with Apyral and Sylodent, while the other five slurries did not differ significantly from each other. Compared to slurry application alone, brushing increased tissue loss (except for Perkasil and Sorbosil 39). This effect was most pronounced with Apyral and Sylodent (each *p* < 0.000 compared to slurry application without brushing). Tissue loss values at the end of the experiment (T4) after slurry application and additional brushing are given in Table 1 and Fig. 1; *p* values for all comparisons between particle types are given in Table 2.

### Detection of Sn on particles

Tin was detectable on the silica particles in the range of 0.6 to 1 wt%, but not on alumina. The respective values are given in Table 3.

**Table 3:** Description of the investigated particle types according to the manufacturer's specifications as well as the amount of tin on the particles after use in F/Sn slurries as determined with EDX in wt % (mean ± SD); n. d.: not detectable; D50: 50% of the particles are smaller than the specified value, D90: 90% of the particles are smaller than the specified value.

Product (name as used in the manuscript)	Particle size (µm)	Particle type	Usage in toothpastes	Sn on particles
Syloblanc 81 (Syloblanc)^a^	Average particle size 5	Hydrated silica	Highly efficient toothpaste abrasive, which possesses a considerable thickening power	0.6 ± 0.2
Sylodent 850c (Sylodent)^a^	Average particle size 15	Hydrated silica	Mild abrasive silica with little thickening capacity	0.7 ± 0.3
Perkasil SM 660 (Perkasil)^a^	Average particle size 16–20	Hydrated silica	Thickener	0.8 ± 0.1
Sorbosil AC 77 (Sorbosil 77)^b^	Average particle size 6–9.5	Hydrated silica	Cleaning, high/medium abrasion	0.8 ± 0.3
Sorbosil AC 36 (Sorbosil 36)^b^	Average particle size 7–14	Hydrated silica	Highly versatile abrasive silica; cleaning, medium/low abrasion	1.0 ± 0.2
Sorbosil AC 39 (Sorbosil 39)^b^	Average particle size 9–14	Hydrated silica	Cleaning, very low abrasion	0.9 ± 0.2
Apyral 24 (Apyral)^c^	D50: 6–9 D90: 18–24	Alumina	Cleaning	n. d

^a^Grace, Düren, Germany; ^b^PQ Corporation, Warrington, England; ^c^Nabaltec, Schwandorf, Germany.

## Discussion

The aim of our study was to investigate the effect of the particle type on the efficacy of toothpaste slurries in reducing erosive and erosive abrasive tissue loss. The types of particles studied were different silica with different abrasiveness, particle sizes and functions in toothpaste formulations, and an aluminium oxide (alumina) particle. The toothpastes were professionally produced by a toothpaste manufacturer, were free of active ingredients and differed only in terms of the particle type. The particle content of toothpastes is commonly between 8 and 20%^4^, thus we have chosen a value of 15%, which is about in the middle of this range. Since the active ingredients were later added during the preparation of the slurries, differences in the formulations, e.g. due to stabilisers, could be avoided. The F/Sn slurries were adjusted to a pH of about 4.5 to prevent precipitation of SnCl_2_. Since the pH value determines the formation of CaF_2_-like precipitates^11^, the pH of the slurries with NaF were also adjusted accordingly. The active ingredient-free slurries were prepared with a neutral pH value to avoid erosive demineralisation.

The null hypothesis that different particle types in experimental toothpastes had no effect on tissue loss values, when applied as slurries only or when applied as slurries with additional brushing, was only partly rejected. Most particle types did not affect the tissue loss, regardless of whether the slurries were applied with or without brushing. Furthermore, when statistically significant effects were observed, they were mostly of minor order of magnitude. Exceptions were Sylodent and Apyral when used in conjunction with F/Sn. In the following, the results are discussed in more detail.

Looking at the results for the slurry application, we found, as expected, only small effects for NaF. What we did not expect, however, was that the type of particle seems to influence the efficacy of the slurry, even if no physical forces are involved. Although the overall differences were not very large, the slurry with Sylodent produced about 20% less tissue loss than the slurry with Sorbosil. The finding that NaF toothpastes of the same fluoride concentration can have very different efficacies^2,3^ could therefore, possibly at least in part, indeed be due to the particle fraction contained.

The F/Sn slurries were much more effective than those with NaF confirming earlier results^1^. However, in the present study, the particle type had a distinct impact on the efficacy of the slurries. For most particle types, after an initial tissue loss, further loss of tissue was even completely inhibited. In the presence of F/Sn, demineralisation processes lead to the incorporation of Sn and possibly fluoride ions into the crystallite structure^12^, so that the enamel then becomes less acid-soluble. This process takes some time, so that initially there is always some tissue loss. This is certainly the reason why F/Sn formulations are often not superior to other fluoride compounds in experiments with initial erosion^10^. The slurry with Apyral was clearly less effective than the five best slurries. Apyral is an alumina which is somewhat soluble under slightly acidic conditions^13^. Alumina is used in other areas to remove fluoride from water^14^, so it can be assumed that aluminium fluoride complexes may have formed in our slurries, reducing the available fluoride. Although SnCl_2_ has an erosion-inhibiting effect on its own, this would seem less than in combination with fluoride^15^. A reduction of available fluoride may therefore explain the lower efficacy of the Apyral slurry, but also the persistent downward drift of the fluoride concentration when measuring the fluoride concentration in the slurry (see below).

Another factor that may influence the active ingredient concentration (and thus the efficacy of F/Sn toothpastes) is the adsorption of Sn to the silica surface. The amount of adsorbed Sn may well vary in particle mixtures of market toothpastes^9^, so we investigated whether the types of particles in our study differed in this respect. However, the amounts of Sn we found in the present experiment were similar for all silica particle types, so the question of why the slurry with Sylodent (which showed relatively good results with NaF but was less effective than the other silica types when combined with F/Sn) remains unclear.

When physical impacts through brushing were added to the system, we had expected that at least in the absence of active ingredients brushing with slurries would produce a higher tissue loss than brushing in water, but there was generally a steady tissue loss in all groups with only minor differences between the particle types. This is in contrast to two other studies with eroded enamel^5,6^ and another with enamel with initial caries^16^, which showed a clear relation between abrasiveness and tissue loss. Erosive demineralisation produces a partially demineralised surface that appears in cross-section as a loosened crystalline structure that is sharply demarcated from the underlying healthy enamel^17^. This demineralised enamel surface has a lower microhardness than sound enamel and the microhardness decreases with increasing erosion. The susceptibility of eroded enamel to brushing abrasion is inversely related to the loss of microhardness and increases over-proportionally with decreasing hardness^18^. It can therefore be speculated that in the present study with relatively severe acid impacts, toothbrushing alone can remove the few micrometres of demineralised surface and that, once sound enamel is reached, no further tissue loss takes place, regardless of whether particle-containing slurries or water were used for brushing. In the two previously mentioned studies^5,6^, a much milder erosion model was chosen, which certainly led to a smaller decrease in microhardness. Under these conditions, the abrasiveness of slurries may have played more a role.

In the presence of NaF, tissue loss was lower in all groups compared to the control. Slurries with the different particle types showed a similar pattern of efficacy as in the experiment without active ingredients. Overall, the differences in tissue loss between the application of slurries alone and the additional brushing abrasion were small, except for the relatively good effect of the Sylodent slurry, which was counteracted by brushing abrasion. Similar results were obtained in our comparisons of market toothpastes^3^, where it was shown that the good efficacy of the slurry had no predictive value for the results under brushing abrasion.

In contrast to these rather small differences between sole slurry application and additional brushing abrasion results in the NaF experiment, brushing with the F/Sn slurries increased tissue loss in most groups compared to slurry application alone. It is well conceivable that the physical effects of brushing disrupt the incorporation of Sn into the crystallite structure, which could explain the further, albeit small, increase in tissue loss up to T3, which was observed for the otherwise very effective slurries with Syloblanc, Sorbosil 36, and Sorbosil 39. The significantly greater tissue loss after application of the slurry with Apyral can be explained by lowering the efficacy of the active ingredients, as discussed above. However, the reduced effect of the Sylodent slurry must also remain unexplained at this point.

Overall, we expected that our experiment would provide an explanation for the varying efficacy of toothpastes with the same active ingredients. However, this was only partially the case, so further studies should also address the question of whether the excipients may have an influence on the efficacy of toothpastes for erosion prevention beyond their basic function (e.g. stabilisation of active ingredients).

The effects of different types of particles on tissue loss are not only of interest for eroded tooth surfaces, but also for those with initial caries, which also exhibit reduced microhardness^19^. Such lesions often occur on smooth surfaces when fixed orthodontic appliances have been used. In contrast to initial caries lesions of proximal surfaces, these so-called white spots are exposed to the physical action of toothbrushes and toothpastes. Accordingly, it has been shown that abrasive toothpastes can erase the surface layer of the lesion, at least in vitro^16^, and the disappearance of white spots after longer observation periods may at least in part be explained by this. Healthy enamel is relatively resistant to physical effects due to its considerable microhardness, at least when neutral toothpaste slurries are used. However, if the pH of a toothpaste slurry is low, tissue loss may well be generated^16^, in which case the role of different particle types would again come into view. All these aspects point to broader perspectives for further research in this context.

A limitation of our study is that the different particles were only investigated with fixed parameters in terms of the excipients in the toothpaste formulation, the particle concentration, the type of brushes and bristles and the erosion/abrasion protocol. All such factors could have a potential influence on the relative effects of the particles studied, so that further research on the topic seems worthwhile.

As a conclusion, it can be stated that the particle types investigated here seem to play a more secondary role for the efficacy of a toothpaste in the context of erosion and erosive wear relative to the type of the active ingredient, at least under the experimental conditions chosen here. Nevertheless, we were able to show at least small differences between the slurries with different silica, so that the efficacy of toothpastes can conceivably be optimised by the selection of particle types. Alumina, on the other hand, seems to be less suitable for F/Sn formulations.

## Methods

Samples were prepared (overall n = 384; n = 16 per group) from previously impacted human third molars stored in saturated thymol (thymol powder; Sigma-Aldrich, Steinheim, Germany) solution until use. Plane-parallel samples (2 mm × 3 mm) were cut from the smooth surfaces and polished (diamond saw and microgrinding system; Exakt-Apparatebau, Norderstedt, Germany; grinding and polishing discs P800, P2400, P4000; Leco, St. Joseph, MI, USA). Depending on the shape of a molar, two or three samples each could be obtained. These were pooled after completion of the sample preparation and randomly assigned to the experimental groups.

The enamel samples were mounted on custom-made metal sample holders (sample carrier according to template; Krose, Cölbe-Schönstadt, Germany). These holders consisted of a central area for fixing the specimens and two parallel reference surfaces 1.6 mm higher, each with four drill marks serving as starting points for repeated profilometric measurements. The parallelism of the reference surface and the sample surface was verified profilometrically.

### Toothpastes and solutions

The toothpastes were experimental formulations provided by a manufacturer (Dr. Theiss Naturwaren; Homburg, Germany). They all consisted of the same basic components (polyethylene glycol, sorbitol, hydroxyethyl cellulose, titanium dioxide, sodium lauryl sulphate, and water). Each experimental toothpaste had a specific type of particle added with 15% w/w (see Table 3).

The mineral salt solution was prepared by dissolving 0.4 g H_3_PO_4_ (orthophosphoric acid 99%; Merck, Darmstadt, Germany) in 40 ml distilled water, 1.5 g KCl (potassium chloride; Sigma-Aldrich, Steinheim, Germany) in 100 ml distilled water and 1 g NaHCO_3_ (sodium hydrogen carbonate; Merck, Darmstadt, Germany) in 100 ml distilled water. All solutions were added together and made up to 600 ml with distilled water. Then 0.2 g CaCl_2_ (calcium chloride anhydrous powder pure; Merck, Darmstadt, Germany) was dissolved in 100 ml distilled water and added to the solution with stirring. The solution was made up to a volume of 1000 ml with distilled water. For the demineralization solution (pH 2.6), 5 g citric acid monohydrate (citric acid monohydrate ≥ 99.5% p.a.; Carl Roth, Karlsruhe, Germany) was made up to 1000 g with distilled water.

Toothpaste slurries (1:3 w/w) were prepared either with the mineral salt solution (no active ingredients, series I) or with solutions containing NaF (series II) or amine fluoride and SnCl_2_ (F/Sn; series III). With reference to the NaF solution, 1.03 g NaF (sodium fluoride; Merck, Darmstadt, Germany) was made up to 1000 g with distilled water. The pH value was adjusted to 4.5 by adding HCl (hydrochloric acid (1 N); Carl Roth, Karlsruhe, Germany). Regarding the F/Sn solution, 9.34 ml amine fluoride (RonaCare Olaflur; Merck, Darmstadt, Germany) was diluted with 250 ml distilled water and then 0.82 g SnCl_2_ (tin(II) chloride dihydrate; Merck, Darmstadt, Germany) was added. The solution was made up with another 250 ml of distilled water. The pH was adjusted to 3.5 by adding HCl (1 N).

The solutions were prepared in order to obtain a theoretical fluoride content of 350 ppm and a Sn content of 875 ppm, which corresponds to a fluoride content of 1400 ppm and a Sn content of 3500 ppm in the undiluted toothpaste. The pH value and the fluoride content of the slurries (mean ± SD) were monitored daily (NaF Slurry: pH 4.6 ± 0.04; 353.5 ± 1.7 ppm F^−^; F/Sn slurry: pH 4.2 ± 0.16; 318.1 ± 20.0 ppm F^−^). In the slurries containing Apyral, no stable reading for the fluoride concentration could be obtained. Instead, after an initial value in the expected order, there was a constant downward drift; after 10 min measuring time, only half of the initial amount of fluoride was found.

### Experimental procedures

The experiment consisted of three series (I, II, and III) of tests with 12 erosion or erosion and abrasion cycles each. One cycle consisted of 6 × 2 min erosion, followed by 6 × 15 min storage in the mineral salt solution. After the first and the sixth erosion had been performed, all sample holders were placed in a container (Färbekasten mit Deckel 42,480; Hecht Assistent, Sondheim vor der Rhön, Germany) with the corresponding slurry for 2 min. During this time, samples in test series I and half of the samples in test series II and III were brushed for 15 s in a toothbrush simulator (brushing machine ZM3; SD Mechatronic, Feldkirchen-Westerham, Germany; ADA reference brush, travel path 6 mm, speed 60, load 200 g, zigzag mode, room temperature), either with one of the test slurries or with water (control). The other half of the samples in test series II and III were placed in the respective slurry for 2 min without brushing. In the test series I, the mere insertion of the samples into the slurries was dispensed due to the absence of active ingredients.

De- and remineralisation procedures were done on a shaker (Schütteltisch 3006; Lauda-GFL, Lauda-Königshofen, Germany; 35/min, 25 °C). The immersion in the slurries was static at room temperature. Before the samples were transferred to any other treatment, they were rinsed for 30 s with tap water.

As control groups in each series, samples were included that were placed in distilled water instead of the slurries and treated as described above with respect to all other applications. In series I, the control group samples were brushed in distilled water for 15 s during each 2 min treatment period. In series II and III the samples were placed in distilled water, half of these samples were additionally brushed, as in the experimental groups.

### Profilometry

The tissue loss was quantified with an optical device (MicroProf; Fries Research & Technology, Bergisch-Gladbach, Germany; sensor FRT CWL F 300 µm). Starting from the drill marks on the reference surface from the sample holder three traces were made at intervals of 0.2 mm and with a length of 4 mm and interpreted with the system software (Mark III). Regression lines were calculated on the sections of the profile that were located on the reference plane and on the sample. Tissue loss (µm) was defined as the vertical distance of these regression lines and was expressed as the mean of the three tracings. Measurements were performed after 3, 6, 9, and 12 cycles (T1–T4).

### Detection of Sn on the particles

F/Sn slurries were centrifuged for 10 min at 10,000 rpm, the sediments were dried for 72 h at room temperature, disintegrated with a soft toothbrush and then applied in thin layers to sample plates. Two sample plates were prepared for each slurry and five particles were selected for energy-dispersive X-ray spectroscopy (EDX) on each sample plate resulting in ten measurements for each F/Sn slurry. A scanning electron microscope (JSM-6510; Jeol, Tokyo, Japan) equipped with a Silicon Drift Droplet Detector (X-Flash Detector 410-M; Bruker Nano, Berlin, Germany) was used, and EDX spectra were obtained at a working distance of 15 mm, a voltage of 15 kV, a spot size of about 60, and a count rate of 1 kcps. The analysis time was 120 s, with a deadtime of ≤ 5%. Tin was determined semi-quantitatively (wt %).

### Statistics

Statistics were done with IBM SPSS Statistics version 25 (IBM Germany, Ehningen, Germany).

The sample size calculation was based on data from a previous study^3^. An α of 0.5 and a β of 0.8 and a relevant difference of 20% from the control group were used. In the data set from^3^, the tissue loss value in the control group was 13.4 with a standard deviation of 2.2; the combined standard deviation in the experimental groups was also 2.2. The sample size calculation basing on these data resulted in a sample size of 12. To compensate for samples that could not be evaluated and measurements of a potentially different magnitude, the sample size was increased by 50% with a total of 18 samples per group. This was the same for the experimental as well as the control groups.

The power analysis of the present data shows that the relevant difference of 20% of the summarised loss values of the control groups can be detected with a power of 1 both with the sole slurry application and with additional brushing (summarised standard deviation 2.0 and 2.3 resp.).

The data were checked for sufficient Gaussian distribution (Kolmogorov–Smirnov test; *p* < 0.05) and presented as mean (± SD). The increase in tissue loss over time within a group was analysed with t-tests for dependent samples. After adjustment for multiple testing (Bonferroni), the significance level was set at 0.002. The comparison of tissue loss after application of the different particle types was made using the data from T4. The comparison of tissue loss from sole slurry application and additional brushing was done with independent samples *t*-tests; after adjustment for multiple testing (Bonferroni), the significance level was set at 0.006. The comparison of particle types was done with ANOVA and Tamhane`s post hoc test.

All tests were two-tailed.

### Accordance with relevant guidelines and regulations

The use of human tissue samples has been approved by the Ethics Committee of the Faculty of Medicine at Justus Liebig University Giessen (Doc. No. 143/09) and informed consent has been obtained from the donors or, where required, from their legal guardians. All methods have been performed in accordance with the relevant guidelines and regulations, if applicable.

## Data Availability

The datasets generated during and/or analysed during the current study are available from the corresponding author on reasonable request.
